# Symptom Management and Quality of Life of Breast Cancer Patients Using Acupuncture-Related Therapies and Herbal Medicine: A Scoping Review

**DOI:** 10.3390/cancers14194683

**Published:** 2022-09-26

**Authors:** Gajin Han, Ye-Seul Lee, Hee Jae Jang, Song-Yi Kim, Yoon Jae Lee, In-Hyuk Ha

**Affiliations:** 1 JINRESEARCH, Namyangju 12113, Korea; 2 Kyung Hee Sweet & Sunny Korean Medicine Clinic, Namyangju 12113, Korea; 3 Jaseng Spine and Joint Research Institute, Jaseng Medical Foundation, Seoul 06110, Korea; 4 Kyung Hee Yakson Korean Medicine Clinic, Suwon 16393, Korea; 5 Department of Anatomy and Acupoint, College of Korean Medicine, Gachon University, Seongnam 13120, Korea

**Keywords:** breast cancer, anticancer treatment, side effects, acupuncture, herbal medicine, scoping review

## Abstract

**Simple Summary:**

This is a scoping review of published literature on the usefulness of acupuncture-related therapies and herbal medicine in alleviating the side effects associated with breast cancer treatments. It is an important study given that the various treatment interventions for breast cancer, such as surgery, chemotherapy, radiotherapy, and hormone therapy, have unpleasant side effects that compromise the patient’s quality of life. The study revealed positive impact of some acupuncture-related therapies and herbal medicines in improving the symptoms and quality of life of patients with breast cancer. These findings will inform further studies on the economic impact of acupuncture and herbal medicines in the management of adverse events in patients on breast cancer treatment.

**Abstract:**

The side effects associated with breast cancer treatments often reduce the patients’ quality of life. The effectiveness of acupuncture-related therapies and herbal medicine in managing the side effect is not fully understood. The study included clinical studies published in the 10 years since 2011 and analyzed the effectiveness of the therapies for managing side effects of anticancer treatment. The databases of MEDLINE via PubMed, CENTRAL, EMBASE, OASIS, and NSDL were searched. Thirty studies, including 13 (43.3%) randomized controlled trials (RCTs), 12 (40.0%) before-and-after studies, three (10.0%) case series, one (3.3%) case report, and one (3.3%) non-RCT, were included in this review. The main symptoms identified were aromatase inhibitors-induced arthralgia (AIA), lymphedema, and chemotherapy-induced peripheral neuropathy (CIPN). The types of acupuncture-related therapies applied included manual acupuncture, electro-acupuncture, moxibustion, and electro-moxibustion. In ten studies, eight herbal medications were administered. The Brief Pain Inventory-Short Form (BPI-SF) and Functional Assessment of Cancer Therapy-General (FACT-G) and -Breast (FACT-B) were frequently used to evaluate pain and QoL, respectively. Most studies suggested beneficial effects of acupuncture and herbal medicine on managing pain, daily function, and quality of life in patients going through AIA, CIPN, and/or lymphedema, with mild side effects. The scoping review implies the potential of CAM therapies as promising interventions for managing symptoms which otherwise lack alternative management options, and for improving the quality of life of breast cancer patients.

## 1. Introduction

Breast cancer is the most prevalent type of cancer and the leading cause of death among women, representing 13.8% of all cancer cases worldwide [1]. In 2018, 23,647 women were diagnosed with breast cancer in Korea, accounting for approximately 20.5% of all cancer incidence among Korean women [2]. Furthermore, in contrast with other types of cancer, such as cancer in the stomach, colon, and rectum, breast cancer incidence in Korea is constantly increasing, and the average annual percentage change is 5.4% [2]. One of the characteristics of breast cancer patients in Korea is the relatively younger age of the patients with an average of 50 years, which is approximately 10 years younger compared to the average age worldwide; in 2018, 13% of breast cancer patients in Korea were still in their 40s [3]. Moreover, the 5-year survival rate of Korean breast cancer patients was 93.3% in 2014–2018 [3,4]. Thus, breast cancer patients in Korea tend to be younger, with more than a 90% chance of 5-year survival, implying a higher rate of return to work (RTW) after or even during cancer treatments [5].

Nevertheless, the side effects of breast cancer treatments, such as surgery, chemotherapy, radiotherapy, and hormone therapy, are exceedingly common and have profound effects on the patients’ quality of life [6]. The symptoms due to cancer treatments include pain, fatigue, sleep disturbances, nausea, vomiting, and depression [7], and some symptoms specifically prevalent in breast cancer patients undergoing treatments include aromatase inhibitor-induced arthralgia (AIA); diarrhea, cough, neutropenia, and myalgia induced by monoclonal antibody therapy (e.g., trastuzumab) [8]; post-surgery symptoms, such as lymphedema [9] and post-mastectomy pain syndrome [10]; and menopausal symptoms such as hot flashes [11]. Recent studies indicate that the consistent use of aromatase inhibitors (AIs) increases the risk of osteoporosis. Many of these symptoms are not fully addressed in Western Medicine, which often leads to unmet needs in breast cancer patients, especially in relation to their health-related quality of life (HRQoL) [12]. Furthermore, the patients’ unmet needs may account for the high usage of complementary and alternative medicine (CAM) therapies, such as acupuncture-related therapies and herbal medicine, by breast cancer patients. Studies reported that approximately half of the patients diagnosed with cancer have experiences with CAM therapies, [13,14] with high levels of satisfaction reported [14].

Several studies have been conducted to examine the effectiveness of CAM therapies in the symptom management of breast cancer patients. Acupuncture and acupressure are suggested for reducing nausea and vomiting [15,16], vasomotor symptoms, such as hot flashes [17,18,19], and cancer-related fatigue [20,21,22]; mind–body therapies, such as meditation and yoga, are suggested for stress reduction and mood disorders [23,24,25]. Based on these findings, several clinical practice guidelines on the use of CAM therapies have been published and updated. A recently updated clinical practice guideline from the Society for Integrative Oncology discusses a variety of adjuvant CAM therapies available to breast cancer patients [25]. In this guideline, acupuncture for nausea and vomiting, hot flashes, and fatigue, and mind–body therapies for stress, anxiety, and depression were recommended; in contrast, ingested dietary supplements were not suggested. Acupuncture to relieve hot flashes was also recommended by the Breast Cancer Clinical Guidelines published by the Northern Cancer Alliance in the United Kingdom to complement the National Institute for Health and Care Excellence and Association of Breast Surgery guidelines [26]. However, many CAM therapies were graded C or D in existing guidelines due to the lack of sufficient evidence [25].

The question that remains to be answered upon examining previous studies is the effectiveness of CAM therapies in symptom management not yet reported in the clinical practice guidelines but still prevalent in breast cancer patients due to anticancer treatments. Although the management of these symptoms is not yet fully elucidated by clinical guidelines and previous studies, they have a strong impact on the patients’ quality of life, as well as on societal costs. A noteworthy update in recent studies is the investigation of the effectiveness of CAM therapies in post-surgery symptoms, such as lymphedema [27,28]; chemotherapy-induced neuropathic pain [29]; and muscle and joint pain due to endocrine therapy, such as AI-related arthralgia [30]. In this regard, the goal of this study is to collect all relevant data on the management of these symptoms using acupuncture-related therapies and herbal medicine and assess the effectiveness of these therapies.

This scoping review aims to provide an update on the efficacy of CAM therapies in symptoms commonly experienced by breast cancer patients associated with surgery and anticancer treatments with no sufficient management options. In this review, our focus is to identify how acupuncture-related therapies and herbal medicine are being used in relieving muscle and joint pain and lymphedema and assess their effectiveness in terms of symptom relief and improvement in HRQoL. The outcome measures used to measure pain and HRQoL, as well as the related results, were collected. Due to the paucity of related publications in the field of the research topic, this scoping review aims to cover two main interventions in CAM, namely acupuncture-related therapies and herbal medicine, at different levels of evidence collected from various types of clinical study designs.

## 2. Materials and Methods

A scoping review design was used to review the current status of studies on acupuncture-related treatments for symptom management and quality of life improvement of breast cancer patients. A scoping review is a method of knowledge synthesis that deals with exploratory research questions aiming to map major concepts, types of evidence, and gaps in research related to a defined area or field by systematically searching, collecting, and synthesizing existing knowledge [31]. This type of review helps researchers, clinical practitioners, and policymakers understand the current status of a particular research area and decide the direction for future research [32].

This study followed the method based on the Arksey and O’Malley framework [32], as well as the Preferred Reporting Items for Systematic Reviews and Meta-Analyses extension for scoping reviews (PRISMA-ScR) [33]. The following five steps were used to conduct this scoping review: (1) identification of the research question; (2) identification of relevant studies; (3) study selection; (4) charting of data; and (5) collecting, summarizing, and reporting the results.

### 2.1. Identifying the Research Questions

Prior to the initiation of this study, the broad research question was ‘how are acupuncture-related therapies and herbal medicine being used in relieving muscle and joint pain and lymphedema in breast cancer patients who underwent anticancer treatments?’

The more detailed research questions used after starting the study were as follows:

1. What kind of research has been conducted?
2. Which acupuncture-related therapies and herbal medicine types are mainly used?
3. What were the main outcomes related to pain and quality of life?
4. What were the results of the treatments?
5. What is the level of evidence regarding the effectiveness of acupuncture-related therapies and herbal medicines?

### 2.2. Identifying the Relevant Studies

The following electronic databases were searched: MEDLINE via PubMed, the Cochrane Central Register of Controlled Trials (CENTRAL), Excerpta Medical dataBASE (EMBASE), Oriental Medicine Advanced Searching Integrated System (OASIS), and National Science Digital Library (NSDL). An example of the specific search strategy is listed in Table 1. Modifications to the search terms and search strategies may be adopted in reference to the database being searched. The initial search was limited to the time period between January 2011 and April 2021, as this scoping review aimed to provide an update on the use of adjuvant CAM therapies. However, the search period in scoping reviews may be subject to change if the collected studies are too few and the authors feel there is a lack of information from the retrieved results.

### 2.3. Study Selection

#### Inclusion/Exclusion Criteria

Human studies including randomized controlled trials (RCTs), controlled clinical trials, case series, case reports, pilot clinical studies, and retrospective observational studies investigating the efficacy and safety of adjuvant acupuncture-related or herbal medicine-related therapies in breast cancer patients were reviewed. To reflect the purpose of this study, this review focused on studies published in the last 10 years, written in English. Existing guidelines, expert consensus papers, and existing qualitative studies focusing on the experience of breast cancer patients in using CAM therapies for the purpose of aforementioned symptom relief were not included in this scoping review; however, they were considered in analyzing the results of this scoping review as complementary information. In vivo and in vitro experiments, reviews, duplicate articles, ongoing studies, and studies that failed to provide detailed results or with incomplete data were also excluded. Table 2 presents elaborate selection criteria for this scoping review.

The eligible participants were defined as breast cancer patients (1) over 18 years of age, (2) who were, or had been, going through anti-cancer therapy, such as breast cancer surgery (either mastectomy or lumpectomy), chemotherapy, immunotherapy, and/or endocrine therapy, and (3) who were experiencing post-surgery pain, neuropathic pain, joint pain, or lymphedema due to anti-cancer therapy. Patients experiencing other types of side effects, such as nausea, vomiting, and hot flashes, were excluded from this scoping review. In addition, clinical studies focusing on the antitumor abilities of acupuncture-related therapies and herbal medicine were not within the scope of this review and were excluded. No restriction was applied on sex, ethnicity, symptom severity, cancer stage, disease duration, clinical setting, and country of study.

Although the definition of CAM therapy may somewhat vary depending on the health care system of each country or the perspective of CAM, this scoping review focused on the interventional role of acupuncture-related therapies and herbal medicine-related therapies utilized by patients as adjuvant therapy to conventional treatments. These CAM therapies included: Traditional East Asian Medicine therapies, such as acupuncture, acupressure, electro-acupuncture, laser acupuncture, massage, and herbal medicine, as described in individual studies. CAM treatments, such as vitamin supplements; mindfulness therapies, such as meditation; and yoga were excluded after thorough discussions among the authors regarding the aim and scope of this review. No limitations were applied to the frequency, dosage, and duration of treatments.

Some examples of the outcomes within the scope of this review include self-reported pain severity; cancer-specific inventory, such as Functional Assessment of Cancer Therapy-Breast (FACT-B), scores; length of hospital stay; patient satisfaction; depression assessment instrument, such as Beck’s Depression Inventory (BDI), scores; absolute circumference or relative volume change of the arm, range of motion (ROM); dosage of painkillers; costs and health-related quality of life assessment tool, such as Short Form (SF)-12, SF-36, and EQ5D, scores. All the outcomes reported in the included studies were listed and mapped during the review process. For example, adjuvant CAM therapies may not be helpful in reducing the absolute circumference of the arm in breast cancer patients suffering from lymphedema, but they might show beneficial effects on HRQoL. Such conflicting results might indicate no benefit from the practitioner’s perspective but imply some benefit from the perspective of the patient facing RTW.

Another dimension that requires attention is the paucity of sufficient measures to address the needs of breast cancer patients. While the anti-cancer treatment regimen of breast cancer is well-designed and presented in the forms of guidelines and clinical pathways, management of side effects of these anticancer therapies is often dealt with limited options. CAM interventions may confer therapeutic effects that are different from those of conventional anti-cancer treatments or usual care. This scoping review assessed the types of outcome indicators to analyze the effects of CAM interventions in the management of side effects. This may also account for the limitations faced by many clinical studies on breast cancer patients using CAM therapies, and the lack of sufficient or proper controls. As this scoping review aims to assess adjuvant CAM therapies, the controls used in clinical studies to compare the effectiveness were assessed with particular interest.

### 2.4. Screening and Agreement

Two authors (G.H. and H.J.J.), who were reviewers trained in the process and purpose of the study, independently searched the electronic databases using the search strategies described above and screened the retrieved studies for eligibility. Upon initial search, all citations were uploaded to EndNote X20 (Clarivate Analytics, Philadelphia, PA, USA). After removing the duplicates, full texts were reviewed in detail against the inclusion criteria. The number of results for each database search was noted and presented in a PRISMA flow diagram. The reasons for excluding studies were recorded for individual studies. All disagreements were resolved by consulting an independent reviewer (Y.-S.L.).

### 2.5. Charting the Data

Study information assessed for this review are as follows: the first author, corresponding author, publication year, country, language, study type, study aim, study population and sample size, patient characteristics (e.g., age, sex, breast cancer stage), surgery history, anti-cancer therapy history, type and cycle of current anti-cancer therapy, randomization method (if applicable), blinding method (if applicable), interventions (type of treatment, number and dosage of administration), type, number and dosage of comparison (if applicable), treatment period, outcome measures, primary outcome, secondary outcome, and statistical analysis method. The third author ((Y.-S.L.) reviewed and confirmed the data extraction process. Any disagreements during the data extraction process were reconciled through discussion or through consulting a fourth author (S.-Y.K.). The data charting table was modified as necessary during the process of data extraction.

### 2.6. Collecting, Summarising, and Reporting the Results

After coding the included studies, the contents of the studies were analyzed. The extracted data were presented in tabular form in line with the objective of this scoping review. The distribution of the studies by the aforementioned extracted information was analyzed. A qualitative analysis was conducted to illustrate an overview of each study. The tabulated results were supported by narrative summaries to describe the results in relation to the objective of this scoping review.

## 3. Results

### 3.1. Literature Search and Selection Process

**Figure 1:**
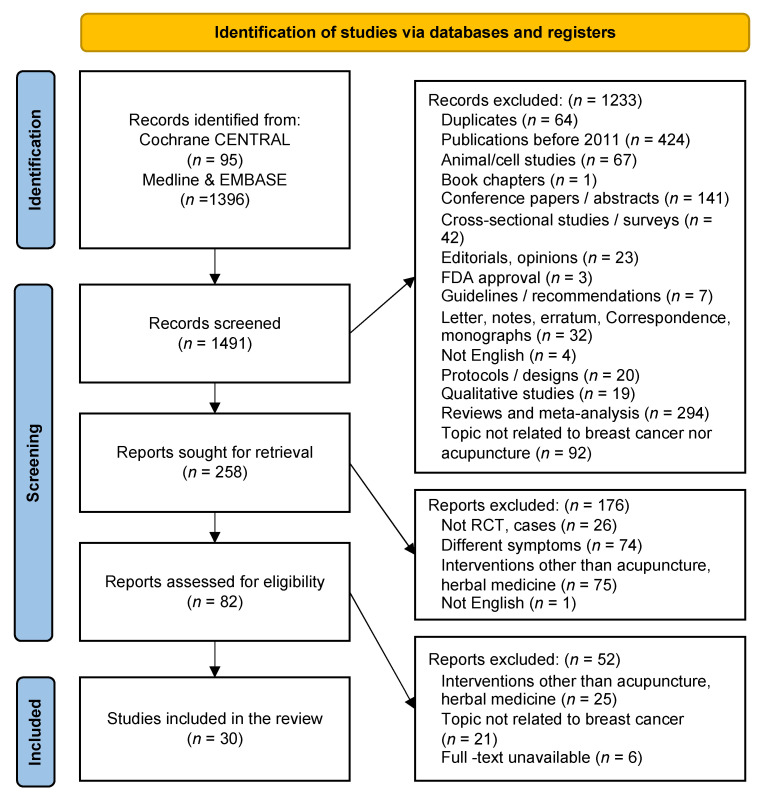
Study Flow Chart.

A total of 1491 studies published between January 2011 and April 2021 were identified. Through the literature selection process, 30 studies were finally selected [29,34,35,36,37,38,39,40,41,42,43,44,45,46,47,48,49,50,51,52,53,54,55,56,57,58,59,60,61,62] (Figure 1). Table 3 summarizes the general characteristics of the included studies. Sixty-four duplicate articles were excluded. Additionally, 1397 studies were excluded based on their titles and abstracts, as they were not related to acupuncture-related therapies for breast cancer, they were not conducted in vivo or in vitro, or they focused on other diseases in patients with breast cancer. Studies without complete results, such as RCT protocols, letters, correspondence, conference abstracts, or RCT protocols, were also excluded. The PRISMA flow chart was used to track the number of articles at each stage of the review. Table 4 elaborates the studies included in this review.

### 3.2. General Characteristics of the Identified Literature

#### 3.2.1. Publication Year

Except for the year 2012, research has been carried out steadily since 2011. The largest number of studies (seven articles) was published in 2018.

#### 3.2.2. Study Design Types

The 30 studies included experimental studies and observational studies. Among the experimental studies, there were 12 before-and-after studies, 13 RCTs, and one non-RCT. Among the observational studies, there were three case reports and one case series (Table 3).

#### 3.2.3. Research Regions

Most studies were conducted in the USA (n = 13), followed by China (*n* = 7), Korea (*n* = 4), Japan (*n* = 1), Taiwan (*n* = 1), Switzerland (*n* = 1), Israel (*n*= 1), Brazil (*n* = 1), and Armenia (*n* = 1) (Table 4).

### 3.3. Characteristics of Study Participants

#### 3.3.1. Participants

A total of 2005 participants were enrolled in the included studies (Table 5). The number of participants in RCTs/non-RCTs or before-and-after studies was 2000. Five patients were the subjects of a case report and three case series. All patients included in this review were female, and no male patients were enrolled in any of the included studies. The age range of patients varied, and only six studies mentioned the duration of breast cancer.

The number of articles focusing on patients with AI-induced side effects was the highest. In addition, there were studies focusing on lymphedema, post-mastectomy syndrome, and chemotherapy-induced peripheral neuropathy (CIPN) (Table 5).

#### 3.3.2. Type of Anti-Cancer Treatments Received by the Patients

Among the articles dealing with the AI-induced side effects, the patients received adjuvant hormonal therapy, such as tamoxifen, anastrozole, letrozole, and exemestane. The patients underwent surgery, chemotherapy, and/or radiotherapy prior to hormonal treatments.

In the studies focusing on patients experiencing breast-surgery-related pain, the patients received total mastectomy, modified radical mastectomy, quadrantectomy, and/or breast-conserving surgery. In addition, the patients in the other studies underwent chemotherapy, such as taxane, oxaliplatin, docetaxel, and paclitaxel administration.

#### 3.3.3. Main Symptoms of Patients

The main clinical symptoms presented in the included articles identified musculoskeletal symptoms (arthralgia and/or stiffness and/or swelling in one or more joints, bone pain, myalgia, carpal tunnel syndrome, trigger finger), lymphedema, neuropathy, post-mastectomy pain syndrome (shoulder and chest wall), fatigue, psychological distress (anxiety, depression), insomnia, vasomotor symptoms (hot flushes and sweating), atypical genital bleeding from vaginal mucosa, sexual dysfunction (vaginal dryness, dyspareunia), mucosal dryness, nausea, vomiting, coping, pruritus, dizziness, and headache (Table 5).

### 3.4. Treatment Details

#### 3.4.1. Acupuncture-Related Treatments

Among the included studies, the number of acupuncture-related articles was 20 (67%). The type of acupuncture-related therapies, such as manual acupuncture (MA), electro-acupuncture (EA), moxibustion, electro-moxibustion, transcutaneous electrical acupoint stimulation (TEAS), contact needle therapy, and auricular acupuncture (AA), varied. There were 10 studies using only MA [43,44,45,49,50,51,52,54,57,59], four studies using only EA [36,37,52,55], one study using MA and EA [29,42], one study using herbal medicine and MA [59], and one study using MA, EA, and AA together [42]. In the study by Lin [60], AA, MA, TENS, and Silver Spike Point therapy were used together. In addition, moxibustion [47], electro-moxibustion [48], and bloodletting puncture and cupping [46] were used in one study each.

All studies except two [52,59] reported the selection of acupoints. Acupoints are specifically chosen sites for acupuncture manipulation according to theories of traditional medicine. A total of 114 acupoints were reported, and the acupoint mostly used was ST36. The acupoints used more than thrice were LI11, LI4, LR3, LI15, SP6, SP9, GV20, KI7, BL60, CV12, CV3, GB21, GB34, LR8, LU5, PC6, SP10, and Baxie. In addition, Ah shi points were also used. Details of acupoints used for each symptom are shown in Table 6.

#### 3.4.2. Herbal Medicine

In 10 of the C, including Sipjeondaebo-tang [34] (Juzentaihoto in Japanese, Shi Quan Da Bu decoction in Chinese), aconitine root [34], oral medication containing *Lens culinaris* lectin [35], tiger bone powder [39], Ikshingungol granules (Ekijingenkotsu in Japanese, Yi Shen Jian Gu in Chinese) [40,41], black cohosh (*Cimicifuga racemosa*) extracts [58], Ikkiyangeumheadok-tang (Ekkiyouingedokuto in Japanese, Yiqi Yangyin Jiedu decoction in Chinese) [59], and curcumin [62]. The type of herbal medicine used included decoction, powder, and granules. Sipjeondaebo-tang was composed of Astragali radix, Cinnamomi cortex, Angelicae radix, Paeoniae radix, Cnidii rhizoma, Rehmanniae radix, Ginseng radix, Atractylodis lanceae rhizome, Poria, and Glycyrrhizae radix. Ikshingungolhwan (Yi Shen Jian Gu granules) was used both in an RCT [40] and a before-and-after study [41] and was composed of Radix rehmanniae Preparata, Fructus Corni, Semen cuscutae, Radix Achyranthis Bidentatae, Cyperi rhizoma, Angelicae Sinensis radix, Poria, Paeoniae Alba radix, Chuanxiong rhizoma, Corydalis rhizoma, and *Phryma leptostachya*. Ikkiyangeumheadok-tang is composed of Astragali radix, *Codonopsis pilosula*, *Poria cocos*, *Glehnia littoralis*, *Curcuma phaeocaulis*, *Pelodiscus sinensis*, *Tulipa edulis*, *Corydalis remota*, *Dendrobium moniliforme*, Ponciri Fructus Immaturus, *Trichosanthes kirilowii*, and *Polygonum multiflorum* [59]. A total of 31 herbal ingredients were used for the prescriptions stated above, and the most used ingredient was *Poria cocos* (three times).

### 3.5. Control

A control group was included in 13 controlled studies and the types of control used were placebo, sham acupuncture, waitlist, usual care, exercise, and kinesiotherapy. Three studies used sham needle as control, two studies used both waitlist and sham controls, two studies used only waitlist control, and three studies used placebo medications as the control.

### 3.6. Evaluation Tools

The main focus of this study was to evaluate the improvement in pain and HRQoL. To evaluate the pain, the Brief Pain Inventory Short Form (BPI-SF) [29,36,40,41,55], Western Ontario and McMaster Universities Osteoarthritis Index [36,40,41], BPI [38], Visual Analog Scale (VAS) [39,46,47,49,50,53], modified BPI (M-BPI) [39], modified Score for the Assessment of Chronic Rheumatoid Affections of the Hands (M-SACRAH) [41], Numerical rating scale (NRS) [42,52,54], shoulder ROM tool [42,48], Neuropathic Pain Scale (NPS) [55], Disabilities of the Arm, Shoulder, and Hand (DASH) questionnaire [51] were used.

FACT-General (FACT-G) [36], M-SACRAH [40,41], FACT-B [39,40,41], European Organization for Research and Treatment of Cancer Breast Cancer-Specific Quality of Life Questionnaire (EORCT QLQ-BR23) [42,48], Measure Yourself Medical Outcome Profile 2 [42], ‘Was it Worth it?’ questionnaire [42,50], FACT-Taxane [55], 36-Item SF (SF-36) health survey [57], Edmonton Symptom Assessment Scale (ESAS) [59], SF-12 health survey [60], EORTC quality of life questionnaire (QLQ) [62], and EORTC Cancer Quality of Life Questionnaire Core (QLQ-C30 version 1.0) [62] were used to evaluate the quality of life.

In addition, the Common Terminology Criteria for Adverse Events (CTCAE) [54,56], Functional Assessment of Cancer Therapy/Gynecologic Oncology Group-Neurotoxicity (FACT/GOG-NTX) scale [54,56], FACT-Neurotoxicity scale [29,55], Neuropathic Pain Symptom Inventory (NPSI) [57], Nerve Conduction Study [57] test results, and Patient Neurotoxicity Questionnaire (PNQ) [29] were used to assess the degree of CIPN.

As for assessing fatigue, the Brief Fatigue Inventory [37], Functional Assessment of Chronic Illness Therapy-Fatigue [42], and revised Piper Fatigue Scale [47] were utilized. In the studies treating lymphedema [33,34,35,36,38,44,45,48], the arm circumference and VAS for swelling were used to measure the improvement in lymphedema. To evaluate the vasomotor symptoms after anti-estrogen therapy, a hot flush symptom diary (severity and frequency) and the Menopause Rating Scale were used [42,58]. In addition, the Pittsburgh Sleep Quality Index [39,42] and the Women’s Health Initiative Insomnia Rating Scale [42] were used for evaluating insomnia. The Hospital Anxiety and Depression Scale [37], VAS [50], and NRS [52] were used to evaluate anxiety. Another study assessed depression by using BDI [51]. In the study [42] by Kim et al., Female Sexual Function Index, Female Sexual Distress Scale, and Arizona Sexual Experiences Scale were used to evaluate sexual dysfunction [42]. In the article by Quinlan-Woodward et al., the NRS was used to evaluate nausea and the ability to cope [52].

Studies also examined changes in serum estradiol and follicle-stimulating hormone levels [39]; bone mineral density [40,41]; blood indices, such as calcium, phosphate, and alkaline phosphatase [41]; cytokine assays (interleukin [IL]-2, IL-4, interferon gamma) [53]; and brain-derived neurotrophic factor, carcinoembryonic antigen, and cancer antigen 15-3 levels. Additionally, the Measure Yourself Concerns and Wellbeing questionnaire (MYCaW) [59], Response Evaluation Criteria in Solid Tumors [62], Eastern Cooperative Oncology Group performance status [62], biothesiometer assessment [55], grooved pegboard test [55], and vibration sensation test [56] were used.

### 3.7. Treatment Effects

1. Acupuncture-related therapies

The pain analyzed in this review was divided into two types: post-mastectomy pain and AI-induced pain. The post-surgical pain was evaluated in one case report, two before-and-after studies focusing on acupuncture [49,50,52], and one RCT focusing on TEAS [53]. Three studies used VAS for assessing postoperative pain [49,50,53], and one study used the NRS for assessing post-mastectomy pain [52]. All four studies reported that postoperative pain improved after acupuncture or TEAS. BPI was utilized for the measurement of AI-induced pain in two RCTs [36,38] and one before-and-after study [42]. There were improvements reported in two of the studies [38,42], whereas no significant difference was demonstrated in the before-and-after study [36].

In contrast, various tools, such as FACT-G [36], EORTC-QLQ [42], SF-36 [57], SF-12 [60], and FACT/GOG-NTX [56], were used in five studies to measure the QoL of patients treated with acupuncture. There were improvements in the aspect of QoL in three studies [42,57,60], whereas there was no significant improvement in one RCT [42] and one before-and-after study [56].

Seven studies compared the arm circumference of patients presenting with lymphedema including three before-and-after studies [41,43,48], one non-RCT [46], and three RCTs [45,47,51]. All studies utilized acupuncture-related therapies for the treatment of lymphedema. In the three before-and-after studies, there was an improvement in the arm circumference after acupuncture [43,44] and electro-moxibustion treatment relatively [48]. In the RCT [45] conducted by Bao et al., acupuncture treatment did not significantly reduce breast cancer treatment-related lymphedema in pre-treated patients receiving concurrent lymphedema treatment, as there was no significant difference between the acupuncture and waitlist group regarding arm circumference or bioimpedance. The remaining RCTs reported that moxibustion [47] and acupuncture with kinesiotherapy [51] significantly decreased arm circumference relatively. In the study [46] by Wang et al., bloodletting puncture and cupping significantly reduced the circumference of the affected arm, and the reduction of arm circumference mainly occurred in the region extending from the wrist crease to 10 cm distal to the wrist crease.

Five studies focused on CIPN, including three before-and-after studies and two RCTs [29,54,55,56,57]. Among these five studies, neuropathic pain was evaluated using the BPI, National Cancer Institute-CTCAE, NPS, NPSI, and PNQ. Except for one before-and-after study [55], the symptoms of CIPN improved after receiving acupuncture-related therapies as reported in one RCT and three before-and-after studies [29,54,56,57].

In terms of CIPN-related QoL evaluation, FACT/GOG-NTX was used in one study by Ogawa et al.; they reported that all patients showed improvement in the FACT/GOG-NTX score after contact needle therapy [54].

2. Herbal medicine

Three studies, including two RCTs [39,40] and one before-and-after study [41], using herbal medicine as intervention compared BPI scores to measure the AIA of patients. These studies presented data showing an improvement in average pain levels, including the degree of worst pain and pain interference.

FACT-B scores were calculated in three studies, including two RCTs [39,40] and one before-and-after study [41], to evaluate the QoL of patients. There were improvements in the aspect of physical well-being and functional well-being in all these studies. However, no significant differences were observed in the FACT-B social/family, emotional, and additional concern subscales in one before-and-after study [41]. In the RCT conducted by Saghatelyan et al., there was no significant difference between the groups in terms of EORTC-QLQ scores [62].

In the case report by Chino et al., a patient with AI-induced atypical genital bleeding and arthralgia was treated with Sipjeondaebo-tang (Juzentaihoto in Japanese, Shi Quan Da Bu decoction in Chinese) and aconitine root [34]. The atypical bleeding and arthralgia disappeared after taking herbal medicines for 5 weeks and 5 months, respectively. However, when the patient discontinued taking herbal medicine, atypical bleeding recurred. Therefore, the patient continued taking the herbal medicine for 2 years without any signs of side effects. In another case report by Zhu, the patient experienced relief from bone pain with improved appetite and gained 11 kg in weight after 8 years of Yiqi Yangyin Jiedu decoction treatments [61]. Another before-and-after study reported that herbal medicine (*Lens culinaris* lectin) improved arthralgia and mucosal dryness [35].

3. Acupuncture combined with herbal medicine

In the case report by Ben-Arye et al., there were improvements in clinical symptoms, such as fatigue, nausea, anxiety, drowsiness, dyspnea, appetite, sleep, well-being, headache, and hot flashes, measured using ESAS and MYCaW [59].

4. Safety

Adverse events (AEs) were reported in thirteen studies. In the before-and-after study by Beuth et al., adverse reactions, such as nausea and bloating, were documented [35]. In the RCT conducted by Mao et al., despite needle placement in the same arm as breast cancer surgery, there were no cases of infection or worsening of lymphedema in the EA or SA groups. However, 18 related AEs were reported by eight participants in both groups. Most AEs reported in the EA group, such as tingling and numbness, were related to the “De Qi” sensation experienced during the acupuncture process [38]. In the study by Li et al., six participants reported stomach discomfort, but the symptoms were tolerable [39]. In the study by Peng et al., 14 participants among those receiving *Yi Shen Jian Gu* granules reported AEs and 16 participants among those receiving placebo granules [40] reported AEs. In the study by Zhang et al., six out of 30 participants reported AEs, such as mild epigastric discomfort, heartburn, hiccup, and mild diarrhoea [41]. In the study by Kim et al., there were six minor self-limiting AEs, such as subcutaneous bleeding and needle pain [42]. In the before-and-after study by Cassileth et al., 12 out of 33 patients reported mild bruising or minor pain/tingling in the arm, shoulder, or acupuncture site at least once. However, there was a transient increase in lymphedema in the axilla of the lymphedematous arm in one patient [43]. In the study by Bao et al., treatment-related AEs, such as bruising, hematoma, and pain, were reported in participants who received acupuncture [45]. In the study by Han et al., one serious AE and six AEs were reported during the trial, but most AEs were irrelevant to the intervention [48]. In the study by Bauml, acupuncture needle site reaction with discomfort, minor swelling, and bruising after acupuncture needle withdrawal was reported [49]. In the study by Bao et al., there were four AEs among 27 patients, concerning mild bruising [56]. In the study by Lu et al., two participants reported mild AEs, such as pruritis in the feet and joint pain [29]. In the study by Rostock, only one AE (nausea) possibly related to the study medication was reported [58].

In contrast, no AEs were reported in seven studies, while the remaining 10 studies did not mention adverse reactions.

## 4. Discussion

This study was conducted to explore the current status of research related to acupuncture-related treatment and herbal medicine treatment in patients with breast cancer through a scoping review. The ultimate aim of this scoping review was to gather information on the use of adjuvant acupuncture-related therapies and herbal medicine to relieve symptoms, particularly pain and lymphedema, caused by ongoing or completed anticancer treatments and assess the effectiveness of acupuncture-related therapies and herbal medicine. The analysis was conducted based on the following five criteria: (1) Types of research conducted; (2) Types of acupuncture-related therapies and herbal medicine applied; (3) Reported outcomes related to pain and quality of life of breast cancer patients; (4) Effectiveness of the CAM treatments employed; (5) The level of evidence regarding the effectiveness of acupuncture-related therapies and herbal medicines.

### 4.1. Main Findings

A total of 30 studies focusing on acupuncture-related therapies and herbal medicine were identified. Studies have been carried out steadily since 2011, culminating in 13 (43.3%) RCTs, 12 (40.0%) before-and-after studies, three (10.0%) case series, one (3.3%) case report, and one (3.3%) non-RCT, with the RCT being the main type of the studies included. The studies analyzed included a total of 2005 female patients presenting with 44 symptoms, such as post-operative chest and/or shoulder pain, arthralgia, lymphedema, neuropathy, vasomotor symptoms, insomnia, anxiety, depression, and sexual dysfunction.

The type of anti-cancer treatments the patients received varied. First, in the studies focusing on postoperative pain, the patients underwent total mastectomy, modified radical mastectomy, quadrantectomy, and/or breast-conserving surgery. The patients received adjuvant hormonal therapy, such as tamoxifen, anastrozole, letrozole, and exemestane, in the studies about AI-induced side effects. The patients in the remaining studies also underwent chemotherapy, such as taxane, oxaliplatin, docetaxel, and paclitaxel.

The treatments analyzed in this scoping review were divided into two main categories: acupuncture-related therapies and herbal medicine. The proportion of acupuncture-related articles was 67%. The type of acupuncture-related therapies included MA, EA, moxibustion, electro-moxibustion, TEAS, contact needle therapy, and AA. A total of 114 acupoints were utilized, and the most frequently used acupoint was ST36. On the other hand, there were 10 studies on herbal medicine among the 30 studies. Eight herbal medicines were administered, including Sipjeondaebo-tang (Juzentaihoto in Japanese, Shi Quan Da Bu Wan in Chinese), aconitine root, oral medication containing *Lens culinaris* lectin, tiger bone powder, Ikshingungol granules (Ekijingenkotsu in Japanese, Yi Shen Jian Gu in Chinese), black cohosh (*Cimicifuga racemosa*) extracts, Ikkiyangeumheadok-tang (Ekkiyouingedokuto in Japanese, Yiqi Yangyin Jiedu tang in Chinese), and curcumin. The types of herbal medicine included decoction, powder, and granules. The aforementioned treatments were administered to breast cancer patients to manage adverse effects from cancer treatments and were not directly involved in anticancer treatments.

Thirteen of the studies included in the review reported adverse events. Seven studies reported no harmful effects, while the remaining 10 studies made no mention of adverse effects. Acupuncture was associated with needle pain, tingling and numbness, subcutaneous hemorrhage, bruising, and mild swelling. Despite the fact that acupuncture treatment for lymphedema was declared safe even in the arm where axillary dissection was performed, one adverse event (AE) warrants caution, since a transient increase in lymphedema was noted, though it resolved quickly. AEs due to herbal medicine were nausea, stomach discomfort, heartburn, hiccup, and mild diarrhoea, most of which were tolerable. In summary, the AEs from complementary therapies of acupuncture and herbal medicine were associated with mild side effects, and in general, safe.

Thirteen RCTs presented a range of control groups, including placebo medications, sham acupuncture, waitlist, usual care, exercise, and kinesiotherapy. There were three studies that used a sham needle as the control, two studies that used both a waitlist and a sham needle as the control, two studies that used only a waitlist as the control, and three studies that used placebo medications as the control. Due to the lack of therapy alternatives for cancer treatment side effects, physicians and patients seeking further support in symptom management may take into account the efficacy of CAM therapies compared to standard care and waitlist. However, verum acupuncture treatment failed to demonstrate superior efficacy compared to active controls such as sham acupuncture in many studies; additional research is still necessary to determine the efficacy of CAM therapies in comparison to sham controls.

Various methods were available for the measurement of the effectiveness of adjuvant therapies to manage AIA, lymphedema, HRQoL, and CIPN. The nature of the outcomes that these methods measure may depend on what the patient or practitioner considers important. Among the patient-reported outcomes (PROs), unidimensional measures such as VAS and NRS, as well as BPI-SF help perceive pain severity intuitively; other PROs such as FACT-G and FACT-B help understand the patient’s functional abilities, and SF-12 helps assess the patient’s quality of life. Outcomes other than PROs include arm circumference, which is an outcome commonly used for lymphedema, and which interestingly was reduced in a number of studies by CAM therapies. On the other hand, levels of E2 and FSH, as well as BMD were not altered by herbal medicine, while symptoms such as arthralgia and AI-induced pain were significantly reduced in the same studies.

### 4.2. Limitations of This Analysis

Our study has two main limitations. First, the definition of intervention is somewhat ambiguous. During the searching stage, we used the search term ‘CAM’. However, we limited the search regarding interventions to acupuncture-related therapies and herbal medicine according to our inclusion criteria in the screening stage. Further reviews with broader scope of CAM therapies may be necessary (ex. Vitamin supplements) to include therapies often used by cancer patients. Second, we did not include the Chinese database, CNKI, which may have led to us missing some studies suitable for this scoping review. Additional studies focusing on the studies in the Chinese database would enable a deeper understanding on the interventions used in mainland China as well as their effectiveness.

## 5. Conclusions

### 5.1. Implications for Research

The results of this study indicate the potential efficacy of acupuncture-related therapies and herbal products for the symptom management of breast cancer patients. Although the level of effectiveness varied between trials, pain management and lymphedema management were demonstrated to be effective across a range of outcome measures. Side effects were generally mild and often not related to the treatments. Further studies are warranted to explore the efficacy of the aforementioned treatments in a larger sample.

The case reports and case series identified in this review provides a summary of data on the effect of acupuncture-related therapies and herbal medicine on symptom management in breast cancer patients. In addition, existing pilot before-and-after studies and RCTs included in the current scoping review indicate that this research area is continuing to develop. Unfortunately, economic data were not included in this scoping review. Studies that focus on the economic data of acupuncture-related therapies and herbal medicine should be conducted in the future.

### 5.2. Implications for Practice

The most important aspect of breast cancer treatment in a Korean Medicine hospital is the management of adverse effects from standard therapies such as chemotherapy, surgery, and radiation. In actual clinical settings, patients’ satisfaction with Korean medicine treatment has been observed to be high. In this review, we demonstrated that acupuncture-related therapies and herbal medicines had promising effects on the symptoms and QoL of breast cancer patients. As stated previously, this outcome is congruent with clinical practice.

In clinical practice, however, patients with lymphedema following breast cancer surgery frequently fear needle insertion in the arm on the side of the surgery site. In this context, the research included in this review demonstrates that acupuncture is a safe and effective treatment option for lymphedema.

## Figures and Tables

**Table 1:** Search Strategy for PubMed.

No.	Search Terms
#1	breast neoplasm [MeSH Terms] OR “breast carcinoma” OR “Neoplasm, Breast” OR “Breast Tumors” OR “Breast Tumor” OR “Tumor, Breast” OR “Tumors, Breast” OR “Neoplasms, Breast” OR “Breast Cancer” OR “Cancer, Breast” OR “Mammary Cancer” OR “Cancer, Mammary” OR “Cancers, Mammary” OR “Mammary Cancers” OR “Malignant Neoplasm of Breast” OR “Breast Malignant Neoplasm” OR “Breast Malignant Neoplasms” OR “Malignant Tumor of Breast” OR “Breast Malignant Tumor” OR “Breast Malignant Tumors” OR “Cancer of Breast” OR “Cancer of the Breast” OR “Mammary Carcinoma, Human” OR “Human Mammary Carcinomas” OR “Human Mammary Carcinoma” OR “Human Mammary Neoplasms”
#2	pain OR “pain management” OR arthralgia OR “aromatase-inhibitor induced arthralgia” OR “aromatase-inhibitor associated arthralgia” OR “post-mastectomy pain syndrome”
#3	Breast Cancer Lymphedema [MH] OR “Lymphedema, Breast Cancer” OR “Breast Cancer Treatment-Related Lymphedema” OR “Breast Cancer Treatment Related Lymphedema” OR “Breast Cancer-Related Arm Lymphedema” OR “Breast Cancer Related Arm Lymphedema” OR “Breast Cancer Related Lymphedema” OR “Postmastectomy Lymphedema” OR “Lymphedema, Postmastectomy” OR “Post-mastectomy Lymphedema” OR “Lymphedema, Post-mastectomy” OR “Post mastectomy Lymphedema”
#4	complementary therapy [MH]
#5	herbal medicine [MH] OR Drugs, Chinese Herbal [MH] OR “herbal medicine” OR herb OR herbs OR herbal OR Medicine, Chinese Traditional [MH] OR “Traditional Chinese Medicine” OR “Traditional Medicine, Chinese” OR “Chinese Traditional Medicine” OR “Chinese Medicine, Traditional” OR Medicine, Korean Traditional [MH] OR “Traditional Medicine, Korean” OR “Korean Traditional Medicine” OR “kampo” OR tang OR decoction OR granule
#6	Acupuncture [MH] OR Acupuncture therapy [MH] OR Acupuncture OR “Acupuncture therapy” OR “Acupuncture Treatment” OR “Acupuncture Treatments” OR “Treatment, Acupuncture” OR “Therapy, Acupuncture” OR “Pharmacoacupuncture Treatment” OR “Treatment, Pharmacoacupuncture” OR “Pharmacoacupuncture Therapy” OR “Therapy, Pharmacoacupuncture” OR Acupotomy OR Acupotomies OR Needling OR Needle OR Electroacupuncture [MH] OR Electroacupuncture OR Acupoint OR Meridian [MH] OR Meridian OR Acupuncture point [MH] OR “Acupuncture point” OR “Body acupuncture” OR “Auricular acupuncture” OR “Ear acupuncture” OR “acupuncture, Ear” [MH] OR “Scalp acupuncture” OR “Intradermal needle” OR “Fire needle” OR “Elongated needle” OR “Warm needle” OR Dry needling [MH] OR “Dry needle” OR “Skin acupuncture”
#7	Moxibustion [MH]
#8	cupping therapy [MH]
#9	((#1 AND #2) OR #3) AND (#4 OR #5 OR #6 OR #7 OR #8)

**Table 2:** The selection criteria of studies for this scoping review.

Criteria	Details
Study type	Human studies including randomized controlled trials, controlled clinical trials, case series, case reports, pilot clinical studies, and retrospective observational studies (In vivo and in vitro experiments, reviews, duplicate articles, ongoing studies, and studies that failed to provide detailed results or with incomplete data were also excluded)
Patients	Breast cancer patients (1) over 18 years of age, (2) who were, or had been, going through anti-cancer therapy, such as breast cancer surgery (either mastectomy or lumpectomy), chemotherapy, immunotherapy, and/or endocrine therapy, and (3) who were experiencing post-surgery pain, neuropathic pain, joint pain, or lymphedema due to anti-cancer therapy
Intervention	adjuvant acupuncture-related therapies (as acupuncture, acupressure, electro-acupuncture, laser acupuncture, massage, moxibustion, and cupping therapy) or herbal medicine (vitamin supplements; mindfulness therapies, such as meditation; and yoga were excluded)
Language	English
Publication Year	Published in the last 10 years
Miscellaneous	No restriction was applied on sex, ethnicity, symptom severity, cancer stage, disease duration, clinical setting, and country of study.

**Table 3:** General characteristics of the final included studies (*n* = 30).

Variables	Categories	*N* (%)
Publication year	2011	2
	2012	0
	2013	4
	2014	5
	2015	1
	2016	4
	2017	1
	2018	7
	2019	2
	2020	3
	2021	1
Location	Korea	4
	USA	13
	China	7
	Japan	1
	Taiwan	1
	Switzerland	1
	Israel	1
	Brazil	1
	Armenia	1
Study type	Case report	3
	Case series	1
	Randomized controlled trial	13
	Before-and-after study (Single arm study)	12
	Non-randomized controlled trial	1

**Table 4:** Summary of included studies.

Study	Location	Design	Intervention	Comparator	Outcomes	Significant Findings	Adverse Event
Chino et al. [34]	Japan	A case study	1. Juzentaihoto 2. aconitine root	none	1. Genital bleeding 2. arthralgia	1. the atypical genital bleeding disappeared 2. the arthralgia almost completely disappeared	There have been no signs of side-effects for 2 years
Beuth et al. [35]	Germany	A single-arm study	Lens culinaris lectin (oral medication containing sodium selenite (300 μg/day), proteolytic enzymes (bromelaine 400 mg/day and papain 400 mg/day) and Lens culinaris lectin (20 mg/day).	none	1. Mean scores of symptoms (arthralgia, mucosal dryness) 2. The severity of side-effects of hormone therapy using scoring system	The severity of side effects of hormone therapy was reduced by complementary treatment. Mean scores of symptoms declined from 4.92 before treatment to 3.16 after four weeks of treatment for arthralgia and from 4.83 before treatment to 3.21 after four weeks of treatment for mucosal dryness, and these were the primary aims of this investigation.	Adverse reactions (e.g., nausea, bloating 12% of patients; 3% stopped the medication) were documented.
Oh et al. [36]	Australia	A pilot randomized controlled trial	Electro-acupuncture (EA)	sham electro-acupuncture	1. Joint pain, stiffness, and physical function were measured with the Western Ontario and McMaster Universities Osteoarthritis Index (WOMAC) 2. Pain severity and interference: Brief Pain Inventory Short Form (BPI-SF) 3. Quality of life (QOL): Functional Assessment of Cancer Therapy-General (FACT-G) 3. Hand strength: grip test 4. a serum marker of inflammation (C reactive protein (CRP))	There were no significant differences in outcome measures. However, positive trends were observed in stiffness and physical function at week 12 in favor of real EA	No serious adverse events were reported during or after acupuncture treatments.
Mao et al. [37]	USA	Randomized controlled trial	Electro-acupuncture (EA)	1. Waitlist 2. Sham electro-acupuncture (SA)	1. Fatigue: Brief Fatigue Inventory (BFI) 2. sleep disturbance: Pittsburgh Sleep Quality Index (PSQI) 3. anxiety, and depression: Hospital Anxiety and Depression Scale (HADS)	Compared to usual care, EA produced significant improvement in fatigue, anxiety, and depression, whereas SA improved only depression in women experiencing AI-related arthralgia.	No mention
Mao et al. [38]	USA	Randomized controlled trial	Electro-acupuncture (EA)	1. Waitlist 2. Sham electro-acupuncture (SA)	1. Pain severity: Brief Pain Inventory (BPI)	Compared to usual care, EA produced clinically important and durable improvement in arthralgia related to AIs in breast cancer patients, and SA had a similar effect. Both EA and SA were safe.	Despite needle placement in the same arm as breast cancer surgery, no case of infection, no reports of development or worsening of lymphedema occurred in either EA or SA groups. Eighteen related adverse events (AEs) were reported by eight subjects in the EA or SA groups during 398 intervention episodes. These AEs were mild in severity and spontaneously resolved without additional medical interventions. The EA group had more adverse events reported than the SA group (16 vs. 4). A major category of AEs reported in the EA group was related to the “De Qi” sensation (N = 6, such as tingling, numbness during the acupuncture process). Both EA and SA groups had similar rates of pain at the needling site (5 and 4 respectively)
Li et al. [39]	China	Randomized controlled trial	Tiger bone powder (Chinese traditional herb)	Placebo	1. Modified Brief Pain Inventory (M-BPI) 2. Visual Analog Scale (VAS) for pain 3. Functional Assessment of Cancer Therapy-Breast (FACT-B). 4. Serum estradiol (E2), follicle-stimulating hormone (FSH) level	1. Reduced pain (M-BPI) -Treatment group : 4.6 → 2.0 -Control group : 4.9 → 5.6 (*p* < 0.001) 2. Reduced pain (VAS) -Treatment group : 6.3 → 3.0 -Control group : 6.4 → 6.6 (*p* < 0.001) 3. Improved QoL -Treatment vs. Control : 22.23 vs. 19.93 (*p* < 0.05) 4. No significant differences were found for the E2 and FSH	Of all 72 enrolled participants, 6 of them (2 in TB and 4 in placebo) reported stomach discomfort, but tolerable. Thus, we suggested to them taking pills half an hour after meals, and no other adverse events were reported. Two participants failed to continue the intervention. One in TB group lost her mother at 3 weeks; thus, she had difficulties in scheduling. Another participant in placebo group, her husband had a job change, and the whole family had moved to Japan on week 8. Other 70 participants had finished the intervention, and there were no lost cases.
Peng et al. [40]	China	Randomized controlled trial	Yi Shen Jian Gu granules (YSJG)	Placebo	1. Brief Pain Inventory Short Form (BPI-SF) 2. Western Ontario and McMaster Universities Osteoarthritis Index (WOMAC) 3. Modified Score for the Assessment and Quantification of Chronic Rheumatoid Affections of the Hands (M-SACRAH) 4. Functional Assessment of Cancer Therapy-Breast (FACT-B) 5. Bone mineral density (BMD)	1. Reduced pain (BPI-SF) -Treatment group : 6.18 → 3.08 -Control group : 6.05 → 4.42 (*p* = 0.001) 2. Improved joint symptoms measured by WOMAC and M-SACRAH 3. Improved QoL 4. BMD showed no apparent change after 12 weeks for both groups	There was a total of 14 participants reporting adverse events among those receiving YSJG granules (33%) and 16 participants among those receiving Placebo granules (39%).
Zhang et al. [41]	China	A single-arm study	Yishen Jiangu Granules (YSJGG)	None	1. Brief Pain Inventory Short Form (BPI-SF) 2. Western Ontario and McMaster Universities Osteoarthritis Index (WOMAC) 3. Modified Score for the Assessment of Chronic Rheumatoid Affections of the Hands (M-SACRAH) 4. Functional Assessment of Cancer Therapy-Breast (FACT-B) 5. Bone mineral density (BMD) 6. Blood indices, such as calcium (Ca), phosphate (P), and alkaline phosphatase (ALP)	1. Reduced pain (BPI-SF) : 5.75 ± 1.87→3.58 ± 2.15 (*p* = 0.002) 2. Improved joint symptoms measured by WOMAC and M-SACRAH 3. Improved QoL (only in physical and functional well-being) 4. Level of BMD, Ca, and P showed no apparent change after 12 weeks for both groups	In the period of this study, 6 out of 30 participants reported adverse events. Three patients reported mild epigastric discomfort, heartburn, and hiccup. Two patients experienced mild diarrhea, and one moderate. All symptoms were alleviated after discontinuing therapy, and symptoms did not appear when patients restarted therapy. There was no dropout because of AEs.
Kim et al. [42]	Korea	A prospective pilot single-arm study	Acupuncture	None	1. Feasibility of the recruitment strategy and enrolment procedure, compliance with the acupuncture sessions and outcome assessments, acupuncture-related adverse events (AEs), and patients’ expectations and experience of acupuncture during the study using Was It Worth It (WIWI) questionnaire 2. Numerical rating scale (NRS) for symptoms for overall symptom severity 3. European Organization for Research and Treatment of Cancer Breast Cancer-Specific Quality of Life Questionnaire (EORCT QLQ-BR23) 4. Measure Yourself Medical Outcome Profile–Version 2 (MYMOP2) 5. Functional Assessment of Chronic Illness Therapy–Fatigue (FACIT-Fatigue) scales 6. Aromatase inhibitor–related knee pain: the Brief Pain Inventory–Short Form (BPI-SF), Western Ontario and McMaster Universities Arthritis Index (WOMAC) 7. Vasomotor symptoms: a hot flush symptom diary (severity and frequency), Menopausal Rating Scale (MRS) 8. Insomnia: Pittsburgh Sleep Quality Index (PSQI-K), Women’s Health Initiative Insomnia Rating Scale (WHI-IRS) 9. Sexual dysfunction: Female Sexual Functioning Index (FSFI), Female Sexual Distress Scale (FSDS) and Arizona Sexual Experience Scale (ASES) 10. Post-mastectomy chest wall or shoulder pain: BPI-SF, Shoulder Range of Motion (ROM) tool	Improved pain (BPI-SF) : 5.0→ 4.3	Six minor self-limiting adverse events were recorded during a total of 78 acupuncture sessions, giving an incidence rate of 8%. These minor AEs consisted of subcutaneous bleeding (*n* = 4) and needle pain (*n* = 2), which were considered to have a causal relationship with acupuncture. There were also other miscellaneous events, including flu-like symptoms (*n* = 6), headache (*n* = 3), facial pain (*n* = 2), fatigue (*n* = 1), dizziness (*n* = 1), diarrhea (*n* = 1), indigestion (*n* = 1), a fall-related injury (*n* = 1), and tingling sensation in the breast (*n* = 1).
Cassileth et al. [43]	USA	A pilot single-arm study	Acupuncture	None	Arm circumference	Arm circumference 4.6 ± 2.2 → 3.7 ± 2.3 (*p* < 0.0005)	During the treatment period, 12 of the 33 patients reported mild bruising or minor pain/tingling in the arm, shoulder, or acupuncture site at least once. One patient experienced a transient (4-day) increase in lymphedema in the axilla of the lymphedematous arm. There were no serious adverse events—no infections or severe exacerbations—after 255 treatment sessions. Similarly there were no treatment-related infections, severe exacerbations, or other serious adverse events during 6 months of follow-up interviews
Jeong et al. [44]	USA	A pilot single-arm study	Acupuncture	None	Arm circumference	There was a significant reduction in the average circumference of the upper arms, the elbow, and the forearms for both the affected and the unaffected limb at the end of treatment (*p* < 0.001, *p* < 0.001, *p* < 0.001 for the affected limb; *p* = 0.027, *p* < 0.001, and *p* < 0.001 for the unaffected limb, respectively).	There were no serious adverse events and no infections or severe exacerbations after 255 treatment sessions and 6 months of follow-up interviews
Bao et al. [45]	USA	Randomized controlled trial	Acupuncture	Waitlist	Circumference and bioimpedance	Arm circumference -Acupuncture group (AC) : 4.74 (2.23) → 4.29 (2.67) -Waitlist group (WL) : 4.82 (2.32) → 4.76 (2.68) Difference between AC and WL : − 0.38 (*p* = 0.14)	No adverse events were reported in the waitlist group at week 6 and no severe adverse events were reported in either arm throughout the course of study. Adverse events were well balanced between the acupuncture group and the waitlist group that crossed over to acupuncture for weeks 6–12. Grade 1 treatment-related AEs, such as bruising (58%), hematoma (2%), and pain (2%), were reported in patients who received acupuncture. Among the 837 acupuncture treatments provided, one possibly related grade 2 skin infection was reported.
Wang et al. [46]	China	A non-randomized controlled trial	Bloodletting puncture and cupping with exercise	Exercise	1. Arm circumference (at the wrist crease, 10 cm distal to the wrist crease, the elbow crease, and 10 cm distal to the elbow crease) 2. Visual analogue scale (VAS) score for pain	1. Bloodletting puncture and cupping significantly reduced the circumference of the affected arm. Arm circumference reduction mainly occurred in the region from the wrist crease to 10 cm distal to the wrist crease -effective rate 95.8% (treatment group) vs. 58.3% (control group) (*p* < 0.001) 2. VAS scores of the two groups were significantly improved after treatment compared with before treatment -Treatment group: 1.30 (1.93) →0.88 (1.20) (*p* = 0.00) -Control group: 0.92 (1.22) → 0.92 (1.35) (*p* = 0.02)	No patient in either group experienced any adverse effects.
Wang et al. [47]	China	A Preliminary Randomized Controlled Trial	Moxibustion	Compression garment	1. Arm circumference (affected arm) 2. Subjective sensation of swelling: visual analogue scale (VAS) 3. Fatigue: Revised Piper Fatigue Scale (RPFS) scores	1. Decreased arm circumference (the difference value in the treatment group was superior to that in the control group) -Moxibustion group: 25.61 ± 2.11→ 24.48 ± 2.02 (*p* = 0.000) -Control group: 26.70 ± 1.93 → 26.09 ± 1.81 (*p* = 0.003) 2. Improved swelling -Moxibustion group: 7.57 ± 1.16 → 4.87 ± 0.87 (*p* = 0.000) -Control group: 7.32 ± 0.89 → 5.41 ± 0.80 (*p* = 0.000) 3. Improved fatigue -Moxibustion group: 4.85 ± 0.79 → 4.43 ± 0.63 (*p* = 0.000) -Control group: 4.87 ± 0.98 → 4.69 ± 0.77 (*p* = 0.000)	No adverse events, such as local burns, bleeding, ecchymosis, or inflammatory reactions, occurred during treatment.
Han et al. [48]	Korea	A single-arm pilot clinical trial	Electronic Moxibustion (EM)	None	1. Differences in Circumferences between Affected Arm and Unaffected Arm 2. Differences in Range of Motion between Affected Arm and Unaffected Arm 3. Changes in Quality of Life Scores using European Organization for Research and Treatment of Cancer QLQ-BR23 (EORTC QLQ-BR23)	1. After 8 weeks of EM treatment, the mean differences in arm circumferences between affected and unaffected upper limbs decreased. 37.10 mm→29.30 mm (*p* = 0.0078) 2. The differences in ROM between affected and unaffected upper extremities improved an average of 8.3° for flexion (*p* = 0.0488) and 3.2° for internal rotation (*p* = 0.0371) 3. Improvement of arm symptoms was significant only at week 5 (*p* = 0.0469).	After 8 weeks of intervention, there were no significant changes in blood analysis and vital signs. One serious adverse event (SAE) and 6 adverse events were reported during the trial, but most were irrelevant to the intervention.
Bauml et al. [49]	USA	A case report	Acupuncture	None	Visual analogue scale (VAS) for post-mastectomy pain syndrome	Resolution of pain VAS: 5 → 0	No mention
Mallory et al. [50]	USA	A single arm study	Acupuncture	None	1. Was-it-Worth-it (WIWI) questionnaire 2. Pain, relaxation, anxiety, tension/muscular discomfort: Visual analogue scale (VAS)	Improved anxiety, tension/muscular discomfort, and pain.	No mention
Giron et al. [51]	Brazil	A randomized controlled trial	Acupuncture with kinesiotherapy	Kinesiotherapy	1. Shoulder range of motion, arm circumference, pain, upper limb function: Disabilities of the Arm, Shoulder and Hand (DASH) questionnaire 2. Depression: Beck Depression Inventory (BDI)	Both groups showed statistically significant improvement of the items assessed: pain, depression, upper limb function, and ADM, and there was no difference between groups	No mention
Quinlan-Wood-ward et al. [52]	USA	A pilot study	Acupuncture	Usual care	1. Pain, nausea, anxiety, ability to cope: Numeric rating scales (NRS)	Acupuncture delivered postoperatively in the hospital after mastectomy can reduce the severity of symptoms experienced, as well as increase the patient’s ability to cope with her symptoms.	No mention
Ao et al. [53]	China	A randomized controlled trial	Transcutaneous electrical acupoint stimulation (TEAS)	Sham transcutaneous electrical acupoint stimulation	1. Pain: Visual analogue scale (VAS) 2. Blood sample collection: Cytokine assays (IL-2, IL-4, IFN-γ)	1. The postoperative VAS scores at T2 and T3 in the TEAS group were significantly lower compared with the sham TEAS group 2. Compared with baseline levels at T0, serum levels of IL-2, IFN-γ and the ratio of IL-2/IL-4 were significantly decreased at T1-T4 in the sham TEAS group	No mention
Ogawa et al. [54]	Japan	A single arm pilot study	Contact needle therapy (CNT)	None	1. Chemotherapy-Induced Peripheral Neuropathy (CIPN): Common Terminology Criteria for Adverse Events (CTCAE), Functional Assessment of Cancer Therapy/Gynecologic Oncology Group -Neurotoxicity (FACT/GOG-NTX) 2. Breakthrough pain: Numerical Rating Scale (0–4) 3. Patients’ objective evaluation	CNT may improve the symptoms of CIPN and associated side effects during the course of chemotherapy and even after a long interval since the last chemotherapy. CNT might be considered one of the safe and effective alternative methods for CIPN.	No mention
Greenlee et al. [55]	USA	A randomized controlled trial	Electro-acupuncture (EA)	Sham electro-acupuncture	1. Pain: Brief Pain Inventory-Short Form (BPI-SF) 2. Quality of life: Functional Assessment of Cancer Therapy- Taxane (FACT-TAX) 3. Neurotoxicity: Functional Assessment of Cancer Therapy-neurotoxicity (FACT-NTX) 4. Peripheral neuropathic pain: Neuropathic Pain Scale (NPS) 5. Sensory neuropathy: biothesiometer 6. Motor neurologic dysfunction: grooved pegboard test	In this randomized, sham-controlled trial of EA to prevent CIPN in women receiving taxane-based chemotherapy for early stage breast cancer treatment, we did not observe differences in pain or neuropathy symptoms between treatment arms at 12 weeks. Unexpectedly, compared to SEA subjects, women on EA experienced greater increases in pain at 4 weeks after taxane completion. No differences were observed between groups with regard to taxane adherence	One adverse event was reported, which was a grade 1 acupuncture needle site reaction with discomfort, minor swelling, and bruising after acupuncture needle withdrawal.
Bao et al. [56]	USA	A single-arm clinical trial	Acupuncture	None	1. Chemotherapy-Induced Peripheral Neuropathy (CIPN): NCI-CTCAE CIPN grade, Functional Assessment of Cancer Therapy/Gynecologic Oncology Group-Neurotoxicity (FACT/GOG-Ntx), Neuropathic Pain Scale (NPS) 2. Vibration sensation test 3. brain-derived neurotrophic factors (BDNF)	Acupuncture was safe and showed preliminary evidence of effectiveness in reducing the incidence of high grade CIPN during chemotherapy	Acupuncture was safe, and tolerable; the only toxicity noted was that four of 27 (15%) patients reported mild bruising.
Jeong et al. [57]	Korea	A prospective single-arm observational study	Acupuncture	None	1. Chemotherapy-Induced Peripheral Neuropathy (CIPN): Neuropathic Pain Symptom Inventory (NPSI), Nerve Conduction Study (NCS) 2. Quality of life: 36-Item Short From Health Survey (SF-36)	Acupuncture improved symptoms of CIPN and QoL in Korean women suffering from peripheral neuropathy after chemotherapy for breast cancer.	All participants were well adapted to acupuncture treatment during the entire treatment period. No serious adverse events were reported
Lu et al. [29]	USA	Randomized controlled trial	Acupuncture	Waitlist	1. Chemotherapy-Induced Peripheral Neuropathy (CIPN): Patient Neurotoxicity Questionnaire (PNQ) 2. CIPN-specific quality of life (QOL): Functional Assessment of Cancer Therapy-Neurotoxicity (FACT-NTX) 3. Neuropathic pain: Brief Pain Inventory-short form (BPI-SF)	8-week acupuncture intervention, versus usual care, led to clinically meaningful and statistically significant improvements in neuropathic sensory symptoms in breast cancer survivors with mild and moderate CIPN after the completion of chemotherapy.	There were no serious adverse events reported in response to the acupuncture intervention in either the immediate acupuncture group or in the waitlist control group. Two participants (one in each group) reported mild reactions that were possibly related to the acupuncture: one developed grade 1 pruritis in the feet, and one developed grade 2 joint pain.
Rostocket al. [58]	Switzerland	A Prospective observational study	Isopropanolic extract of black cohosh (1–4 tablets, 2.5 mg)	None	Menopause rating scale (MRS II)	The reduction of the total MRS II score under black cohosh treatment from 17.6 to 13.6 was statistically significant.	Only one adverse event (nausea) was possibly related to the study medication.
Ben-Arye et al. [59]	Isarael	case report	Cimicifuga racemosa herbal capsules & Acupuncture	None	1. Quality of life: Edmonton Symptom Assessment Scale (ESAS) 2. Headache, hot flashes, well-being: Measure Yourself Concerns and Wellbeing questionnaire (MYCAW)	Improved fatigue, nausea, anxiety, drowsiness, dyspnea, appetite, sleep, well-being on ESAS scores and headache, hot flashes and well-being on MYCAW scores	No mention
Lin et al. [60]	Taiwan	single arm prospective test	Acupuncture	None	1. Quality of life: Medical Outcomes Study 12-Item Short-Form Health Survey (SF-12) 2. Patient satisfaction questionnaire	Most patients were satisfied with the program. SF-12 showed improvement significantly at the end of study.	No serious adverse effect was reported.
Zhu [61]	China	Case report	Yiqi Yangyin Jiedu Decoction	None	Symptoms of debilitation, waist pain, restlessness at night, loss of appetite, constipation, dry mouth, and bitter mouth	The patient has no bone pain with good appetite and spirit, and gained 11 kg in weight. The treatment has a good clinical effect for eight years	No mention
Saghatel-yan et al. [62]	Armenia4. BMD showed no apparent change after 12 weeks for both groups	Randomized controlled trial	Curcumin (CUC-1^®^, 300 mg solution, once per week)	Placebo	1. Objective response rate (ORR) by Response Evaluation Criteria in Solid Tumors (RECIST) 2. Physical condition (PC): ECOG performance status 3. Patient-reported quality of life (QOL): European Organization for Research and Treatment of Cancer (EORTC) quality of life questionnaire (QLQ), patient’s self-assessment questionnaire (QLQ-C30 version 1.0) 4. Carcinoembryonic antigen (CEA) and cancer antigen 15-3 (Ca 15-3)	Curcumin in combination with paclitaxel is efficacious in the treatment of advanced and metastatic breast cancer.	No mention

**Table 5:** Demographic and clinical characteristics of patients.

Study	Sample Size (Male/ Female) (If RCT, Randomized)	Age (Treatment /Control)	Disease Duration	Main Symptoms	Type of Anticancer Treatment			Target Disease
AI side effects								
Chino et al. [34]	1(0/1)	55	9 years	atypical genital bleeding from vaginal mucosa and joint pain of bilateral hands and knees	total mastectomy Chemotherapy anastrozole			AI side effects
Beuth et al. [35]	680 (0/680)	58.3	Unknown	mucosal dryness and arthralgia	adjuvant hormone therapy			AI side effects
Oh et al. [36]	32(0/32)	<45 12 (86)/14 (93) ≥ 45 2 (14)/1(7)	≤5 years 11 (79)/13 (87) >5 years 3(21)/2(13)	pain and/or stiffness in one or more joints	Aromatase inhibitors			AI side effects
Mao et al. [37]	76(0/76)	59.7	Unknown	fatigue, sleep, and psychological distress	Aromatase inhibitor			AI side effects
Mao et al. [38]	67(0/67)	57.5 ± 10.1/60.9 ± 6.5/60.6 ± 8.2	Unknown	Arthralgia	Aromatase inhibitors			AI side effects
Li et al. [39]	72(0/72)	55(27–73)/ 52(31–72)	Unknown	Pain, quality of life	Adjuvant chemotherapy Adjuvant taxane Aromatase inhibitors (Anastrozole Letrozole Exemestane)			AI side effects
Peng et al. [40]	84(0/84)	57.3(46–74) /59.3(43–76)	Unknown	musculoskeletal symptoms (arthralgia and/or stiffness and/or swelling in one or more joints, bone pain, myalgia, carpal tunnel syndrome, trigger finger)	Aromatase inhibitors Prior tamoxifen Anastrozole Letrozole Exemestane			AI side effects
Zhang et al. [41]	30(0/30)	59.3 ± 8.4	Unknown	Arthralgia Bone pain Myalgia Morning stiffness Carpal tunnel syndrome Trigger finger	Aromatase inhibitor Anastrozole Letrozole Exemestane			AI side effects
Kim et al. [42]	8(0/8)	40–49: 2 50–59: 5 60–69: 1	Unknown	AI-related arthralgia (particularly knee pain), Vasomotor symptoms, including hot flushes and sweating after anti-estrogen therapy, Insomnia, Sexual dysfunction (vaginal dryness, dyspareunia), Post-mastectomy pain of the chest wall or shoulder	aromatase inhibitors, five were taking anti-estrogen agents, two were receiving sedatives and two were taking analgesics			AI side effects, General pain
Rostock et al. [58]	50(0/50)	56 (43–77)	8.6 (+ 6.2) month	climacteric complaints	Tamoxifen			AI side effects
Lin et al. [60]	45(0/45)	53.3 ± 8.3	Unknown	Fatigue, arthralgia, nausea, and insomnia	hormonal therapy (Tamoxifen, Anastrozole)			AI induced symptoms
Lymphedema								
Cassileth et al. [43]	37(0/37)	55(55/65)	Unknown	Upper-limb lymphedema	breast-cancer surgery			Lymphedema
Jeong et al. [44]	9(0/9)	58.44 ± 7.21	Duration of lymph-edema (month) 67.44 38.12	lymphedema	breast-cancer surgery			Lymphedema
Bao et al. [45]	82(0/82)	65(54–71)/ 58(49–70)	Duration of lymph-edema 2.5/2.2	lymphedema	breast-cancer surgery			Lymphedema
					Type of breast cancer surgery			
					Lumpectomy	10 (25%)	11 (26%)	
					Mastectomy	30 (75%)	31 (74%)	
					Type of axillary surgery			
					Sentinel lymph node biopsy	2 (5%)	0 (0%)	
					Axillary lymph node dissection	37 (93%)	42 (100%)	
					Unknown	1 (2%)	0 (0%)	
Wang et al. [46]	75(0/75)	59.90 ± 7.02/ 56.96 ± 5.33	Unknown	lymphedema	breast-cancer surgery Breast conserving surgery (right) Breast cancer modified radical mastectomy (right) Radical mastectomy (right) Total mastectomy (right) Breast conserving surgery (left) Breast cancer modified radical mastectomy (left) Total mastectomy			Lymphedema
Wang et al. [47]	48(0/48)	59.42 ± 7.02/58.25 ± 6.19	Unknown	Lymphedema, Fatigue	breast-cancer surgery Breast conserving surgery (right) Breast cancer modified radical mastectomy (right) Total mastectomy (right) Breast cancer modified radical mastectomy (left) Total mastectomy (left)			Lymphedema
Han et al. [48]	10(0/10)	53.0 (45.0–60.0) /-	Unknown	Lymphedema (arm circumference, shoulder range of motion), Quality of life	breast-cancer surgery			Lymphedema
Post-mastectomy symptoms								
Bauml et al. [49]	1(0/1)	47	4 years	postmastectomy pain syndrome, fatigue, depressed mood	breast-cancer surgery			Post-mastectomy pain
Mallory et al. [50]	20(0/20)	Unknown	Unknown	anxiety, tension/muscular discomfort and pain	mastectomy and/or breast reconstruction			Post-mastectomy pain
Giron et al. [51]	48(0/48)	53.7 ± 11.1 (total)	Unknown	Lymphedema, (Pain, arm circumference, shoulder range of motion) depression	breast-cancer surgery Quadrantectomy Mastectomy Immediate reconstruction			Post-mastectomy pain
Quinlan-Wood-ward et al. [52]	30(0/30)	53.7± 9.4/62.5± 11.5	Unknown	Pain, Nausea, Anxiety, and Coping	mastectomy			Post-mastectomy pain
Ao et al. [53]	70(0/70)	45.6 ± 9.8/46.9 ± 8.6	Unknown	Pain, postoperative nausea and vomiting, pruritus, dizziness and headache	mastectomy			Post-mastectomy pain
Zhu [61]	1	51	11 years	Debilitation, waist pain, restlessness at night, loss of appetite, constipation, dry mouth, and bitter mouth	Breast surgery			General symptoms
Chemotherapy-Induced Peripheral Neuropathy (CIPN)								
Ogawa et al. [54]	6(0/6)	64.3	Unknown	Pain, quality of life	Chemotherapy (taxanes and oxaliplatin)			Chemotherapy-Induced Peripheral Neuropathy
Greenlee et al. [55]	63(0/63)	51.8 ± 10.7 /48.3 ± 12.0	Unknown	Pain, quality of life	Taxane			Chemotherapy-Induced Peripheral Neuropathy
Bao et al. [56]	109 (0/109)	47(39–53)	Unknown	Pain, quality of life	paclitaxel			Chemotherapy-Induced Peripheral Neuropathy
Jeong et al. [57]	10(0/10)	58.7 ± 7.5	Unknown	Pain, quality of life	Taxane			Chemotherapy-Induced Peripheral Neuropathy
Lu et al. [29]	40(0/40)	54.0(32.0–68.0) /53.5(36.0–71.0)	17.3 (1.4–92.0) month/13.3 (5.3–92.4) month	Pain, quality of life	Taxane			Chemotherapy-induced Peripheral Neuropathy
Chemotherapy induced symptoms								
Ben-Arye et al. [59]	1(0/1)	27	Unknown	hot flashes, insomnia, quality of life	palliative chemotherapy with docetaxel			Chemo-therapy induced symptoms
Saghatel-yan et al. [62]	150 (0/150)	57.59/54.17	Unknown	Quality of life	adjuvant or neoadjuvant chemotherapy			Chemo-therapy induced symptoms

**Table 6:** Summary of the intervention (acupuncture-related therapies) in the included studies.

Study	Intervention	Acupoint	Frequency	Treatment Period
Oh et al. [36]	Electro-acupuncture	Day 1: LI4, LI11, GB34, ST40, LR3, GV20, Shishencong, Baxie Day 2: GB21, TE5, ST36, SP6, LR3, GV20, Shishencong, Baxie pain and stiffness of the arms and hands: LI11, LI4, GB21, TE5, the Baxie extra point pain and stiffness of the legs and feet: GB34, ST36, ST40, SP6, LR3 stress levels and cognitive function: GV20, Shishencong. LR3 immune function: ST36, LI4	1.20 min, 2.alternating frequencies of 2–10 Hz	1. twice weekly for 6 weeks 2. total, 12 sessions
Mao et al. [37]	Electro-acupuncture	A Shi point and at least four distant points (the acupuncturist chose at least four local points around the joint with the most pain. Additionally, at least four distant points were used to address non-pain symptoms, such as depression/anxiety and fatigue, that are commonly seen in conjunction with pain)	1. 30 min 2. 2 Hz	1. twice a week for two weeks, then weekly for six more weeks, for a total of ten treatments over eight weeks 2. total, 10 sessions.
Kim et al. [42]	Manual acupuncture & Electro-acupuncture & Auricular acupuncture	1. AI-related arthralgia (particularly knee pain MA: LI4, LR3, GB39, SP6 EA: SP10-SP9, ST34-ST36, GB33-GB34 2. Vasomotor symptoms, including hot flushes and sweating after anti-oestrogen therapy MA: GB20, HT7, LR3, PC6, SP6, SP9, ST36 EA: BL23-BL32 AA: Shenmen, Internal secretion 3. Insomnia MA: Sishencong, GV20, HT7, PC6, SP6, BL62, KI6 AA: Shenmen 4. Sexual dysfunction (vaginal dryness, dyspareunia) MA: PC6, ST30, ST36, SP6, LI4, LR3, CV2, CV4 EA: BL32-BL33 AA: Shenmen 5. Post-mastectomy pain of the chest wall or shoulder MA: LI4, LI11, PC6, ST36, HT7 plus Ah shi points EA: Jiaji (point selection based on dermatomes affected by the lesion) AA: Shenmen, Internal secretion	1. 20 min 2. Low-frequency (2 Hz) and high-intensity (nearly 80% of the patient’s pain threshold) stimulation was used for EA	1. giving a total of 8–12 sessions during the 4-week study period
Cassileth et al. [43]	Manual acupuncture	TE14, LI15, LU5, CV12, CV3, LI4, ST36, SP6	1. 30 min 2. rotation of the needles with lift and thrust	1. twice weekly for 30 min over 4 consecutive weeks 2. total, 8 sessions
Jeong et al. [44]	Manual acupuncture	KI10, LR8, LU8, LR4, HT8, LR2, KI2, LU5, LR8, LR1, SP3, SP2, HT9, HT3, HT7, LU10, SP1, SP5, SP9, LU9, KI7, KI3, KI1, PC9, PC3, PC7, PC8, BL66, LI1, SI5, BL60, LI2, GB43, GB44, GB38, GB41, ST36, ST41, SI3, SI2, SI8, LI11, ST44, BL67 BL40, BL65, TE3, TE2, TE10, TE6 (identifying a pattern of each patient’s symptoms and signs, target meridian and acupuncture points were selected for each patient. Basic combination formulas of Saam acupuncture were determined according to the pattern, which is relevant to one of four syndromes)	1. 30—5 min at each session. 2. manipulated manually to obtain De Qi	1. 6 weeks of the treatment with 3 acupuncture sessions per week 2. total, 18 sessions
Bao et al. [45]	Manual acupuncture	CV12, CV3, TE14, LI15, LU5, LI4, ST36, SP6	1. lasted 30 min 2. De-qi sensation was achieved at certain acupoints, such as LI4 and ST36	1. twice a week for six consecutive weeks. 2. total, 12 sessions
Wang et al. [46]	Bloodletting puncture and cupping	LI14, LI13, LU6, HT2, TE5, SI9, PC3, or ashi points (the most swollen areas or subcutaneous nodules) on the affected arm	1. 15 min,	1. every 5 days for 15 min/session
Wang et al. [47]	Moxibustion	LI14,LI13, TE5, SI9, BL23, and any Ashi points	1. 30 min	1. 4 consecutive weeks. 2. 30 min every 2 days
Han et al. [48]	Electronic Moxibustion	LI14, LI11, TE5	1. 30 min	1.8 consecutive weeks 2. twice per week for 8 weeks
Bauml et al. [49]	Manual acupuncture	distal points: GV20, LI4, SP6, BL60	1. 20 min~30 min 2. needling manipulation until de qi sensation was achieved.	1. weekly for eight treatment sessions.
Mallory et al. [50]	Manual acupuncture	GV20, EX-HN3, LI4, LI11, PC6, SP10, SP6, ST36, LR3, GB40	De-qi sensation	No mention
Giron et al. [51]	Manual acupuncture	CV3, SP9, ST36, KI7, LR3, GB21, LI15, HT14, LU5, LI 4, ST 38, BL 60	30 min	1. once a week for 10 weeks 2. 10 sessions
Quinlan-Woodward et al. [52]	Manual acupuncture	No mention	36 min	No mention
Ao et al. [53]	Transcutaneous electrical acupoint stimulation (TEAS)	LI4, PC6, ST36	1.30 min 2. dense-and-disperse frequency of 2/100 Hz	1. TEAS was performed for 30 min prior to the induction of anesthesia. 2. Postoperative TEAS was performed for 30 min each time at 4 and 12 h post-surgery on the day of surgery, and administered three times (8 a.m., 2 p.m. and 8 p.m.) daily at postoperative days 1 and 2.
Ogawa et al. [54]	Contact needle therapy	1. Points for all patients: CV12, CV4, ST25, KI2 2. Selected points: LR8, LR14, SP3, LR13, LU9, LU1, KI7, GB25, PC7, CV17, CV6, CV4, ST36, LU1, BL20, BL13, BL18, BL23	30–60 s	4–6 sessions
Greenlee et al. [55]	Electroacupuncture	GB34, ST36, LI4, LI10, L3, L5, Ba Feng, C5, C7, Ba Xie	1. 30 min 2. 2 Hz of mixed pulsatile intervals 3. De qi sensation	1. 16 sessions 2. once a week for 16 weeks
Bao et al. [56]	Manual acupuncture & Auricular acupuncture	MA: LI4, TE5, LI11, ST40, Ba Feng AA: shen men, point zero, and two additional auricular acupuncture points where electrodermal signal was detected	30 min De qi sensation	No mention
Jeong et al. [57]	Manual acupuncture	LI 4, LI 11, ST 36, LR3, M-UE-9 (Ba Xie), M-LE8 (Ba Feng)	1. 25 ± 5 min at each session. 2. gently manipulated manually to obtain De Qi,	1. 8 weeks 2. 4 weeks of acupuncture treatment and 4 weeks of follow-up after the last treatment. 3. 3 times a week during the first 4 consecutive weeks.
Lu et al. [29]	Manual acupuncture (MA) & Electroacupuncture (EA)	MA: Yin Tang, LI11, SP9, ST36, K3, Qiduan EA: TE5, Baxie, SP6, LR3	1. De Qi sensation 2. alternating 2–10 Hz 3. 30 min each session	1. 18 sessions of acupuncture over 8 weeks,
Ben-Arye et al. [59]	Acupuncture & Herbal medicine	No mention	15 min	No mention
Zhu et al. [60]	auricle acupuncture & manual acupuncture & Electro-acupuncture & transcutaneous nerve stimulation (TENS) & Silver Spike Point (SSP)	face: S7 shoulder: GB21, LI15, SI11 upper extremities: LI11, LI10, TE5, LI4 lower extremities: SP10, ST36, SP9, KI7	1. 2 and 100 Hz, alternatively	1. 6 sessions 2. 2 to 3 times per week
